# Supplementary material for: Dipeptidyl Peptidase 4 Stimulation Induces Adipogenesis-Related Gene Expression of Adipose Stromal Cells
Source: Int J Mol Sci. 2023 Nov 8;24(22):16101. doi: 10.3390/ijms242216101 (PMC10671339; doi:10.3390/ijms242216101)
Supplement: Supplementary file 1 [file ijms-24-16101-s001.zip › ijms-2643103-supplementary.pdf]

**Dipeptidyl peptidase 4 stimulation induces adipogenesis-related gene expression of adipose stromal cells**

Hsiao-Chi Lai[1,2], Pei-Hsuan Chen[1,2], Chia-Hua Tang[1], Lee-Wei Chen[1,2,3*]

## Supplemental Figures

**Supplemental Figure 1:** MCP-1-supplemented control plasma increases the protein expression of adiponectin and DPP4 in SVFs from adipose tissue of *Lepr*$^{db/db}$ mice. Uncropped Western blot images of pNF-κB, NF-κB, pJNK, JNK, adiponectin, DPP4 and β-actin of SVFs from adipose tissue of *Lepr*$^{db/db}$ mice. Representative images and statistical analysis are presented in Figure 3A.

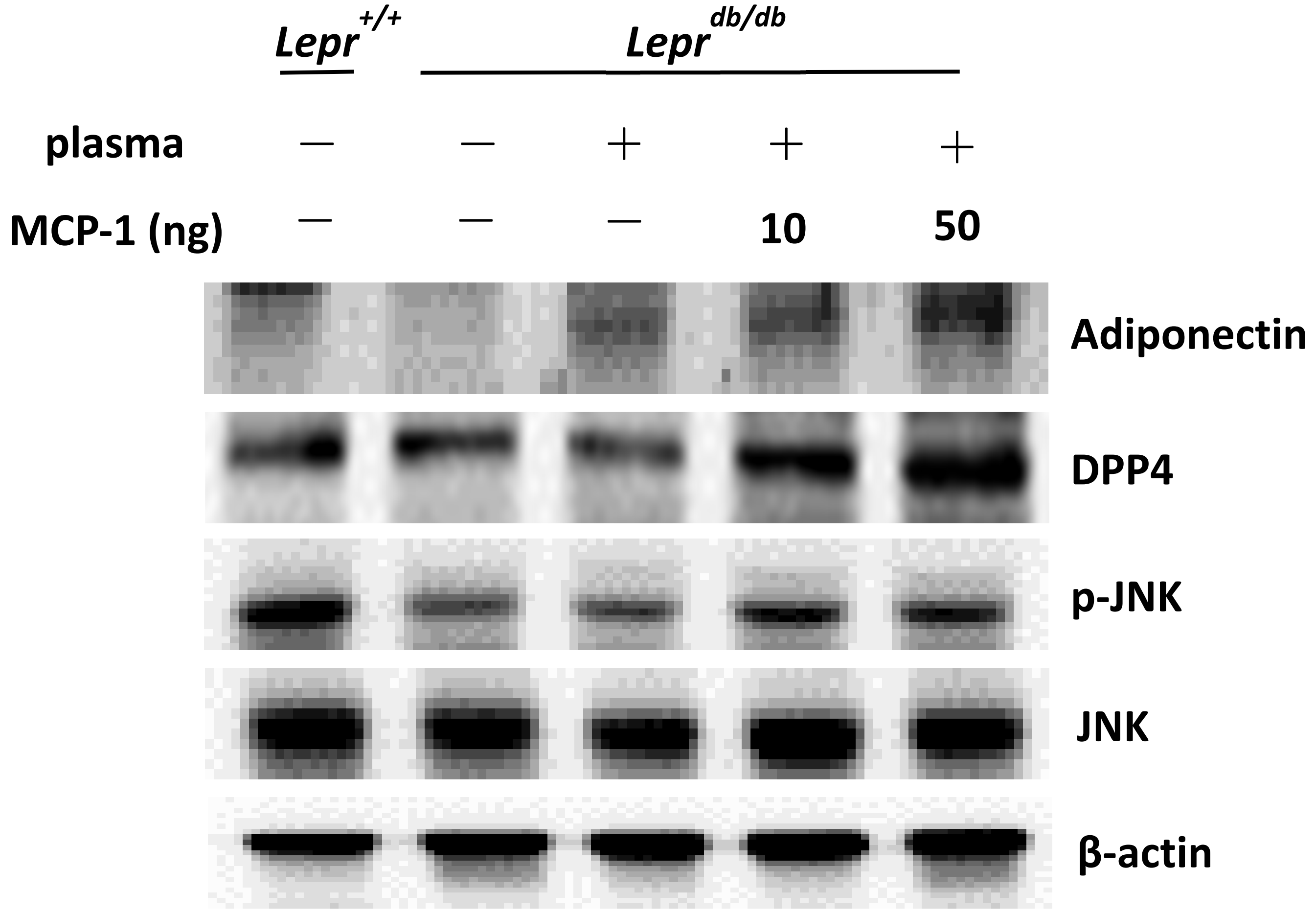

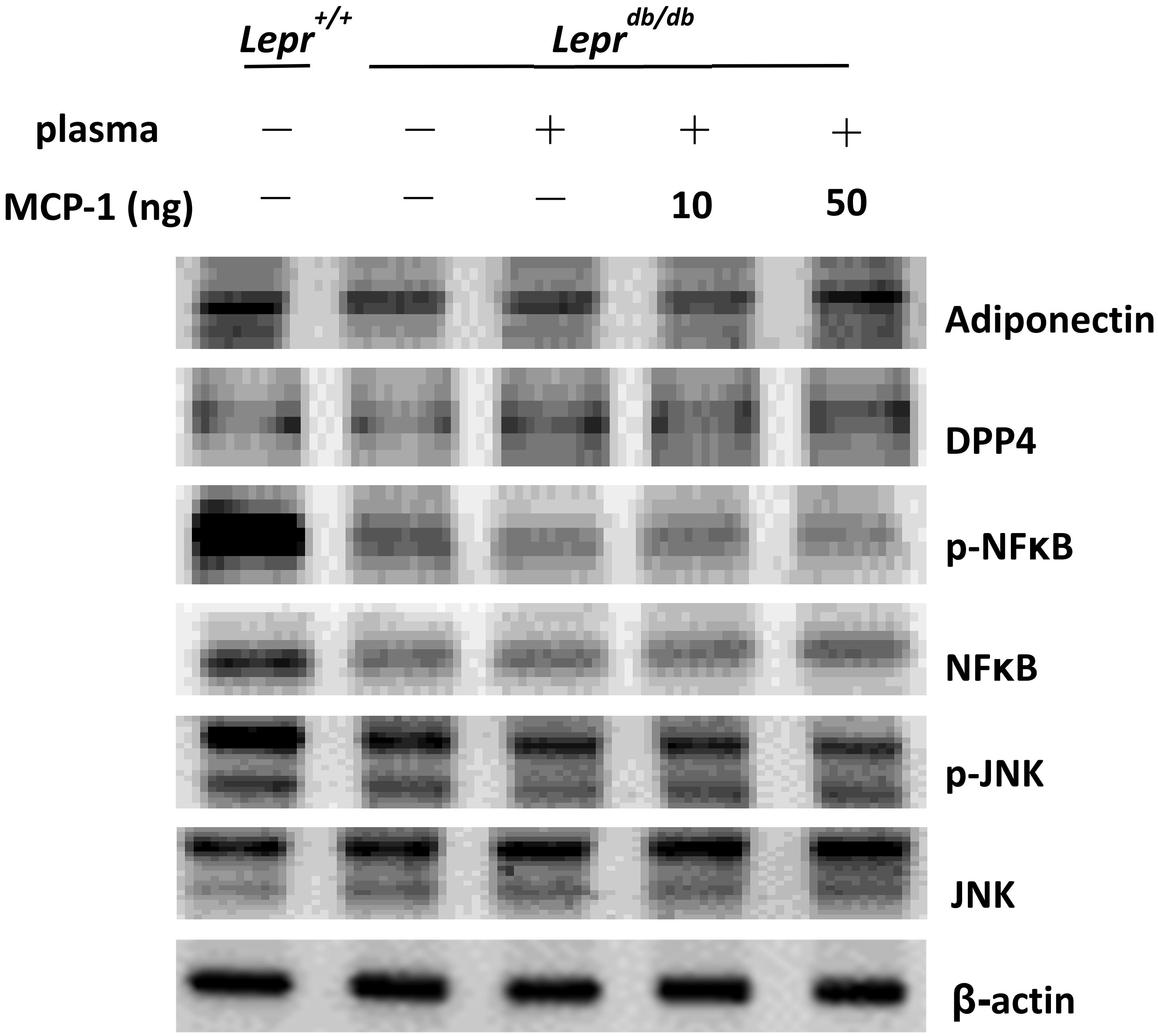

Lepr+/+
Leprdb/db
plasma
—
—
+
+
+
MCP-1 (ng)
—
—
—
10
50
Adiponectin
DPP4
p-NFκB
NFκB
p-JNK
JNK
β-actin

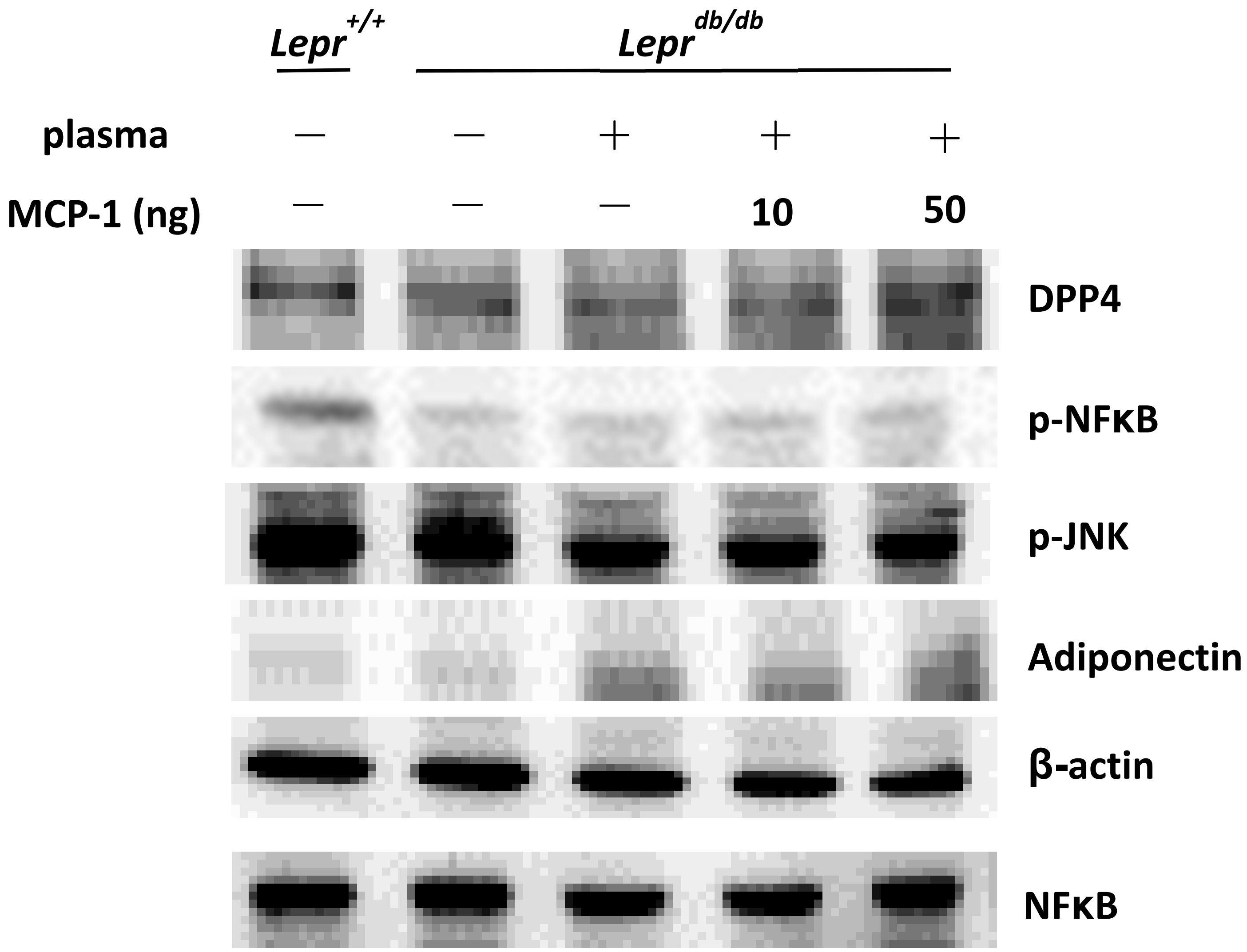
Lepr+/+
Lepr db/db
plasma
MCP-1 (ng)
10
50
DPP4
p-NFκB
p-JNK
Adiponectin
β-actin
NFκB

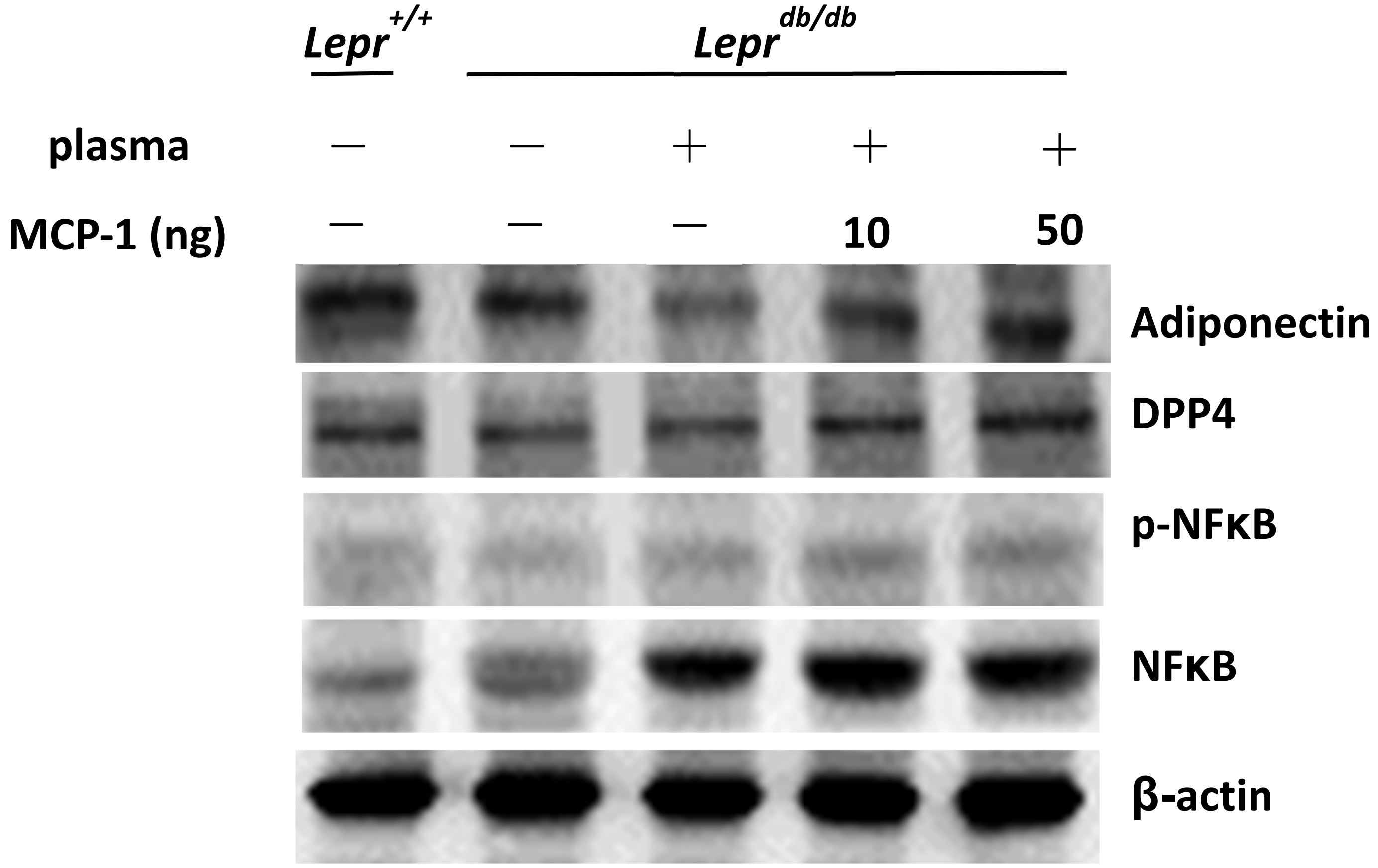

Lepr+/+
Lepr db/db
plasma
MCP-1 (ng)
10
50
Adiponectin
DPP4
p-NFκB
NFκB
β-actin
